# Physiological Evidence and Therapeutic Outcomes of Vitamin D on Cardiovascular Diseases

**DOI:** 10.2174/011573403X263417231107110618

**Published:** 2024-01-18

**Authors:** Abolfazl Zendehdel, Amir Shakarami, Ehsan Sekhavati Moghadam

**Affiliations:** 1 Department of Geriatric Medicine, Ziaeian Hospital, Tehran University of Medical Sciences, Tehran, Iran;; 2 Department of Cardiology, Faculty of Medicine, Lorestan University of Medical Sciences, Khorramabad, Iran;; 3 Department of Cardiology, Ziaeian Hospital, Tehran University of Medical Sciences, Tehran, Iran

**Keywords:** Vitamin D, cardiovascular, physiological functions, myocardial infarction, heart failure, hypertension, parathyroid hormone

## Abstract

Vitamin D hormone is an important regulator of various physiological functions, and its deficiency is characterized by an imbalance in parathyroid hormone and calcium homeostasis. The role of vitamin D in cardiovascular physiology is well demonstrated in animal and human-based studies. In this context, hyperlipidemia, increased atherogenic plaques, cardiac inflammation, hypertension, myocarditis, myocardial infarction, and heart failure are some of the commonest known conditions connected with vitamin D deficiency. Supplementation of vitamin D is recommended to achieve normal serum vitamin D concentrations, nonetheless, in clinical trials often seen discrepancies concerning the supplementation effects and effectiveness. This review summarizes the data on the role of vitamin D in cardiovascular health along with some recent clinical findings regarding the effects of vitamin D supplementation.

## 1 INTRODUCTION

Vitamin D, a micronutrient that is metabolized to a secosteroid hormone (calcitriol), is primarily synthesized in the skin upon exposure to UV radiation (290–315 nm). The process involves the conversion of 7-dehydrocholesterol into pre-vitamin D3 which then transforms into vitamin D3 (cholecalciferol) through isomerization, mediated by the temperature changes [1]. Figs. (**1** and **2**) provide an overview of the metabolism of vitamin D. Notably, vitamin D exists in two main forms: 25-hydroxyvitamin-D (25(OH)D) with a half-life of about 2-3 weeks, and its active metabolite, 1,25-dihydroxy-vitamin D (1,25[OH]2D) detected within 4-6 of its synthesis Serum levels of 25(OH)D serve as a key indicator of vitamin D status due to its extended half-life, stability, and higher concentrations [2]. Excess of vitamin D is catabolized into inactive metabolites by the action of 24-hydroxylase. Serum levels of parathyroid hormone and calcium regulate this feedback mechanism [3, 4]. Dietary sources of vitamin D include fish, meat, liver, kidney, and eggs [1]. Vitamin D2 (ergocalciferol] is produced by the UVB-facilitated metabolism of ergosterol in plants and fungi and is also utilized for fortification [5].

According to the guidelines of the Endocrine Society, serum levels of 25(OH)D < 20 ng/ml are defined as vitamin D deficiency, levels of 20-30 ng/ml as insufficiency, levels 30-50 ng/ml as the normal range, and levels > 50 ng/ml as possibly harmful [6]. About 30-50% of the world’s population is estimated to suffer from vitamin D deficiency.

However, beyond these biological foundations, Vitamin D deficiency arises from a complex interplay of factors that go beyond mere biological characteristics such as skin pigmentation [7] and geographic location [8]. While these attributes undoubtedly shape the body's capacity to synthesize vitamin D through exposure to UV radiation, a comprehensive understanding must encompass various other significant determinants. Among these factors, lifestyle choices play a pivotal role. Individuals who spend a considerable amount of time indoors, don clothing [7] that covers most of their skin or consistently employ high-SPF sunscreen [7] may inadvertently curtail their skin's exposure to the essential UVB radiation, which is vital for optimal vitamin D production. Moreover, advancing age [7] can accentuate the risk of vitamin D deficiency. As individuals grow older, the skin's ability to synthesize vitamin D upon sun exposure diminishes. Beyond sun-dependent factors, dietary habits [8] also significantly influence vitamin D levels. While certain foods like fatty fish, liver, egg yolks, and fortified products contain appreciable amounts of vitamin D, inadequate consumption of these nutrients can lead to deficiency, particularly among those adhering to vegetarian or vegan diets. Also, certain medical conditions affecting the digestive system can hinder the absorption of vitamin D, exacerbating deficiency risk. Gastrointestinal disorders, such as celiac disease [9], Crohn's disease [10], or even obesity [11], can compromise the body's ability to absorb dietary vitamin D efficiently.

Interestingly, the prevalence of vitamin D deficiency varies across different regions. For instance, Europeans and Caucasians have shown a lower prevalence of vitamin D deficiency, while regions like India, Pakistan, Afghanistan, and Tunisia are known to have the highest frequency of vitamin D deficiency [12].

The primary function of vitamin D is to regulate the levels of calcium, phosphate, and minerals for various bodily functions. A deficiency of vitamin D is characterized by increased parathyroid hormone levels and a reduction in bone density. Severe vitamin D deficiency often causes osteomalacia and rickets [13]. Additionally, vitamin D deficiency is linked to numerous pathologies, including cardiovascular [14], gastrointestinal [inflammatory bowel disease] [9], metabolic [15], skeletal [16], and dermatological [17] conditions, as well as allergic diseases [18] and respiratory infections [19-22].

A large number of studies have shown that vitamin D deficiency is associated with a greater risk of cardiovascular morbidity and mortality [23], associated with dyslipidemia [24], atherosclerosis, and hypertension [22, 25]. A recent meta-analysis concluded that vitamin D deficiency might be associated with an increased risk of myocardial infarction [26]. Other biomarkers associated with CVD, glutamyl transferase (GGT), uric acid, and hemoglobin A1c (HbA1c) are also more increased in vitamin D deficient individuals. Associations of low vitamin D status and increased risk of cardiovascular diseases are also seen in patients with diabetes type II and renal disease, together with reduced glomerular filtration rate and bone mineral density, and increase in CRP, glucose, and HbA1c and total cholesterol to high-density lipoproteins (HDL)-cholesterol ratio [27]. Conversely, in patients with diabetes type I, it was shown that 1,25(OH)2D supplementation had some cardioprotective effects such as reduction in ventricular wall thickness, improvement of ejection fraction, end diastolic and systolic volume, and fibrosis [28, 29].

Interestingly, Hansen *et al.* [30], showed an association between both high and low levels of vitamin D and cardiovascular morbidities in type I and II diabetic patients. The study showed that vitamin D levels higher than 50 mg/ml and lower than 10 mg/ml were characterized by a greater incidence of cardiovascular autonomic neuropathy, due to suppressed parasympathetic activity.

This comprehensive review adeptly synthesizes the wealth of data elucidating the role of vitamin D in cardiovascular well-being, encompassing contemporary clinical insights into the impacts of vitamin D supplementation.

## 2 METHODS

We conducted a comprehensive review of the existing literature to explore the intricate interplay of factors contributing to vitamin D deficiency and its implications for cardiovascular health. Our search strategy encompassed various reputable databases, including Scopus, EBSCO, PubMed, and others, to ensure a thorough examination of relevant studies. The search was not restricted by publication date and encompassed research articles, reviews, and clinical studies.

Given the narrative nature of this review, we focused on qualitative synthesis rather than employing a systematic review or meta-analysis approach. This allowed us to present a comprehensive overview of the topic, analyzing and summarizing findings from various studies to provide a holistic understanding of the subject matter. Through a critical evaluation of the selected literature, we aimed to highlight the multifaceted connections between vitamin D deficiency and cardiovascular health.

## 3 VITAMIN D RECEPTORS AND THE CARDIOVASCULAR SYSTEM

There is a wide expression of vitamin D receptor (VDR) and vitamin D metabolizing enzymes (1-α hydroxylase and 24-hydroxylase) across many tissues [23]. VDR, a member of nuclear receptor subfamily 1, group I, forms a heterodimer with retinoid-X-receptor (RXR), upon activation with vitamin D, which binds to VDR-response elements in nuclear DNA (located on the promoter region of the target genes) [31]. The VDR–RXR heterodimer complex bound to VDR-response elements regulates the expression of many genes in the cells and the activation of second messenger pathways [32].

Additionally, in rats with chronic renal failure, VDR activation increased the cardiac levels of miRNA 29b and 30c and decreased expression of their target genes collagen I (COL1A1), MMP-2, and connective tissue growth factor (CTGF), which are the indicators of cardiac fibrosis [33, 34]. Animal modeling of cardiac steatosis presented with myocardial infarction and heart failure reduced VDR activity causes increased expression of diacylglycerol acyltransferase 1, atrial natriuretic, and B-type natriuretic peptides, collagen 1a, and 3a, osteopontin, matrix metalloproteinase (MMP) and tissue growth factor-beta (TGF-β) thereby, increasing lipotoxicity, fibrosis, and hypertrophy of cardiac myocytes.

There is also a role of VDR in heart immunoregulation. VDR modulates the expression of T helper Th1 and Th2 cells in the heart, and the absence of VDR signaling in VDR-/- mice leads to an increase in CD4+ Th2 response and interleukin 4 (IL-4) transcripts, thereby increasing inflammation [35]. In accordance, in the hearts of patients with myocarditis who undergo heart transplantation, a lower expression of VDR, as well as an increase in Th2 cells and Th2 cytokines was shown [35].

A recent study has reported that altered expression of VDR-target genes in the aortic adventitia, like overexpression of growth arrest and DNA-damage-inducible protein 45 alpha (GADD45A) and nuclear receptor corepressor 1 (NCOR1), and lower expression of paraoxonases 2 (PON2), is connected with the greater risk of rheumatoid arthritis in coronary artery disease patients [36]. These findings are in parallel with the role of vitamin D in inflammatory pathologies such as arterial wall atherosclerosis. Reduced VDR expression is also seen in ventricular arrhythmias, mediated by a decrease in heat shock protein-70 (HSP-70) and increased renin-angiotensin 1 levels [37].

Li *et al.* [38] reported that the upregulation of long noncoding RNA (lnRNA), CASC15, in hypertrophic cardiomyocytes is mediated by the increased expression of VDR, where the promoter region of CASC15 had a strong affinity for the VDR complex. This leads to decreased expression of miRNA-432-5p and the upregulation of TLR4 (toll-like receptor 4) mRNA. Hence, they concluded that the upregulation of VDR is among the contributing factors of cardiac hypertrophy, pronounced by the increased expression of brain natriuretic peptide (BNP), beta-myosin heavy chain (β-MHC), and atrial natriuretic factor (ANF). VDR expression is important for vascularization since it promotes the proliferation of pericytes and endothelial cells during retinal vascular development [39].

Furthermore, in an animal study, Tianbao *et al.* [32] showed that VDR is upregulated in reperfused ischemic myocardium. These mice, when treated with calcitriol, showed a reduction in apoptosis, oxidative stress response, mitochondrial dysfunction, and infarction size whereas, improvement in left ventricular function was seen. Therefore, it was concluded that VDR, when stimulated with the agonist, has cardioprotective effects.

## 4 VITAMIN DASSOCIATED POLYMORPHISMS AND CARDIOVASCULAR EFFECTS

Polymorphisms in VDR are reported to alter several significant biochemical processes, including cardiovascular ones. The most common of these polymorphisms are ApaI (rs7975232), BsmI (rs1544410), FokI (rs2228570), and TaqI (rs731236). A recent study has shown that VDR FokI polymorphism with CC genotype is associated with a greater risk of hypertension [40]. Similarly, Sun *et al.* [41] reported that rs3847987 single nucleotide polymorphism with AA genotype is characterized by increased levels of triglycerides and fasting blood glucose in the Chinese population. Moreover, rs2189480 AA genotypes were reported to be significantly associated with the greater prevalence of cardiovascular diseases, particularly in female subjects and individuals aged 55 years and above. In a recent Chinese study, the significantly lower levels of vitamin D and altered presence of VDR variants -an increased prevalence of heterozygous and minor alleles for FokI (f minor allele) and TaqI (t minor allele) variants, were found in patients with heart failure [42].

According to the results from the Egyptian case-control study in mothers and neonates, maternal FokI f genotype variation in the VDR gene is not only connected with lower levels of maternal vitamin D but also with the increased odds of cyanotic congestive heart failure in neonates [43]. Additionally, FokI polymorphism, with the A allele being the pathological allele, has been reported to relate to increased total cholesterol, low-density lipoprotein (LDL) – cholesterol, and decreased HDL- cholesterol levels. These conditions are likely to be more prevalent in cardiovascular patients with rheumatoid arthritis [44]. PaI GT polymorphism was also associated with higher lipid levels and greater body fat [45]. Similar findings have been reported for TaqI and BsmI variants.

BsmI polymorphism with a heterozygous and homozygous B allele variant is also associated with the increased risk of cardiovascular anomalies in chronic kidney disease patients [45, 46]. Similar findings have been reported for TaqI and BsmI variants [47]. However, seems that cardiometabolic risk factors in vitamin D-deficient patients are chiefly mediated by increased adiposity [48], even though discrepancies have been reported in this regard [49].

Additionally, FokI polymorphism, with the A allele being the pathological allele, has been reported to relate to increased total cholesterol (TC), low-density lipoprotein (LDL) – cholesterol, and decreased HDL- cholesterol levels. These conditions are likely to be more prevalent in cardiovascular patients with rheumatoid arthritis [48]. ApaI GT is also associated with higher lipid levels and greater body fat [45]. Similar findings have been reported for TaqI and BsmI variants [47]. Cardiometabolic risk factors in vitamin D deficient patients are chiefly associated with adiposity, however, discrepancies have been reported in this regard [50].

Polymorphisms in vitamin D metabolizing enzymes such as CYP1A1 (rs4646421) and CYP1B1 (rs2551188 and rs1056836) have also shown significant association with hypertension and vitamin D deficiency, particularly in geriatric patients [51].

## 5 VITAMIN DMEDIATED IMMUNE RESPONSE AND CARDIOVASCULAR DISEASE

One of the most significant non-classical actions of vitamin D is the regulation of the immune system. A wide range of innate and adaptive immune responses are regulated by the action of vitamin D. 1,25(OH)_2_D downregulates macrophagic response by reducing the expression of Toll-like receptors (TLR) 2, 9 and 4, respectively, thereby repressing the overall inflammatory response [39]. Down-regulation of TLR 9 reduces the production of IL-6 [52]. Downregulation of c-Jun N-terminal kinase 2 (JNK2) signaling pathway in macrophages also facilitates anti-inflammatory response *via* the action of vitamin D. Active vitamin D downregulates macrophage JNK activation, thereby decreasing inflammation and suppressing oxidized LDL cholesterol uptake and foam cell formation, while deletion of JNK2 prevents vitamin-D-deficiency-induced hypertension and atherosclerosis in mice [53]. The M2 phenotype of macrophages also produces TGF-β and IL-10, thus having anti-inflammatory effects in tissues. They also promote tissue remodeling and scar repair. Studies have shown that these modulators of M2 macrophages are likely to have therapeutic effects on inflammatory diseases, such as atherosclerosis [54]. Vitamin D is the best-known immunomodulator in this case. Gunasekar *et al.* [55] reported that TGF response and neointimal formation are greater with the greater expression of M1 macrophages in epicardial adipose tissue of atherosclerotic swine, while vitamin D supplementation reduced the levels of M1 macrophages and increased the levels of M1 macrophages. Additionally, vitamin D sufficiency was associated with a reduction in monocyte chemoattractant protein 1 (MCP1), tumor necrosis factor α (TNF-α), C-C chemokine receptor type 7 (CCR7), and marker CD14 in epicardial adipose tissue. Furthermore, dendritic cells (also knowns as antigen-presenting cells) express VDR, where vitamin D mediates decreased production of IL-12 and increased production of IL-10, the later promoting T cell regulatory (Treg) response [56, 57]. T and B lymphocytes also express VDR. The adaptive role of vitamin D is characterized by the suppression of the production of interferon-gamma (INF-γ) from Th1 and IL-4 from Th2 cells. It also regulates Th17 and Treg cells and induces the production of immunoglobulins (Ig) from B cells [58].

In accordance, low levels of vitamin D in neonates contribute to the greater thickness of intima-media, a biomarker for subclinical atherosclerosis [59]. Rasa *et al.* [60] in their recent study reported the cardioprotective effects of vitamin D against coronary artery disease and atherosclerosis. Patients without any vascular defect had higher vitamin D levels along with higher levels of anti-inflammatory cytokines [TGF-β and IL-35). Reduced levels of TGF-β, IL-35, and vitamin D were associated with the severity of the disease, whereas higher levels of vitamin D were also found significantly associated with increased TGF-β in these patients. High levels of C-reactive protein (≥ 0.2 mg/dL) and vitamin D deficiency are also reported in individuals with cardiovascular diseases [61]. Additionally, since cyclic AMP has been reported to stimulate renin expression, the effect could be also conveyed by the downregulation of cAMP-protein kinase, thus inhibiting the binding of the cAMP response element-binding protein to the promoter region of the renin gene, as shown by a study *in vitro* [62].

## 6 FROM ANIMAL TO HUMAN-BASED STUDIES

In animal models, vitamin D treatment has revealed protective effects against various cardiovascular pathologies. Martoell *et al.* [63] showed that the treatment with 1,25(OH)2D3 decreased the formation of angiotensin-II–induced dissecting abdominal aortic aneurysm in apolipoprotein E knockout mice, which was associated with reduced inflammatory response, cytokine production (MMP-2, MMP-9, CCL2, CCL5, and CXCL1) and leukocyte infiltration and neo vessel formation [64]. This was accompanied by decreased phosphorylation of extracellular signal–regulated kinases (ERK1/2), p38 mitogen-activated protein kinase (p38-MAPK), and nuclear factor-κB (NF—κB). Additionally, the VDR–RXR interaction in the aortas of 1,25(OH)2D3-treated mice was increased [63]. In another study, vitamin D treatment in streptozocin-induced diabetic mice reduced the diabetes-induced endothelial dysfunction and apoptosis in endothelial cells, which was accompanied by the suppression of prolyl isomerase-1 (Pin1), IL-1β, IL-6, malondialdehyde (MDA), reactive oxygen species (ROS) and upregulation of nitric oxide (NO) and superoxide dismutase (SOD) levels [65]. In an additional study in streptozotocin-induced apolipoprotein E deficient (Apoe-/-) mice, both 1,25(OH)_2_D_3_ and RXR agonist (bexarotene) reduced the expression of nuclear factor-kappa B (NF-κB) and nicotinamide adenine dinucleotide phosphate (NADPH) oxidase, as well as the progression of atherosclerosis and indexes of oxidative stress and inflammation (MDA, IL-6), while increased the expression and levels of SOD [66]. The combined therapy alleviated atherosclerosis, endothelial apoptosis, oxidative stress, and inflammation markers to a greater extent than either monotherapy [67]. In mice with induced MI by ligating the left coronary artery, vitamin D treatment led to the improvement in left ventricular systolic function, less left ventricular wall thinning, and inhibition of cardiac interstitial cell proliferation, which was associated with a trend for reduced myocardial fibrotic area after MI [68, 69].

## 7 VITAMIN D SUPPLEMENTATION AND CARDIOVASCULAR EFFECTS IN HUMANS

Vitamin D supplementation can influence the cardiovascular system by modulating RAS, hyperparathyroidism, insulin resistance, dyslipidemia, hyperglycemia, and inflammation. The renin-angiotensin system (RAS) is a known regulator of blood pressure and other cardiovascular functions. Some studies have proposed that vitamin D is involved in the RAS imbalance since VDR knockout mice have overactivated RAS and developed hyperreninemia, hypertension, and cardiac hypertrophy [70]. Renal tissues of mice lacking VDR (as well as human podocytes with silenced VDR) displayed downregulation of Human Silent Information Regulator Type 1 (SIRT 1), which increased expression of p53, angiotensinogen, renin, and angiotensinogen II type 1 receptor, leading to the increased activation of RAS.

Studies have shown that vitamin D deficiency is highly prevalent in hypertensive patients, probably due to the hyperactivation of RAS and increased levels of parathyroid hormone [71]. Treatment with 50 000 IU/week of oral cholecalciferol for 8 weeks in hypertensive patients with low levels of vitamin D restored normal levels of vitamin D, followed by decreased plasma renin activity, renin, angiotensin II, and improvement in the endothelial function characterized by the increase in flow-mediated dilation. However, no effects on systolic and diastolic blood pressure were shown in a recent meta-analysis in patients with diabetes type 2 [72]. Vitamin D supplements can influence the cardiovascular system by modulating hyperparathyroidism, dyslipidemia, hyperglycemia, and inflammation. Elevation in the levels of parathyroid hormone, a consequence of vitamin D deficiency, is also associated with increased cardiovascular risks [73]. A recent meta-analysis of randomized control trials in type 2 diabetic patients concluded that vitamin D might be beneficial in lowering parathyroid hormone, CRP, LDL- cholesterol, and increasing HDL- cholesterol levels. Interestingly, a study by Ostadmohammadi *et al.* [74] concluded that vitamin D supplementations in type 2 diabetics could additionally decrease fasting sugar, insulin concentration, and insulin resistance (assessed by homeostatic model - HOMA-IR and quantitative insulin-sensitivity check index -QUICKY), while the study of Swart *et al.* [72] showed the opposite, that in the subgroup with very low baseline vitamin D levels, vitamin D supplementation increased glucose and insulin concentrations. Additionally, the results from a recent clinical trial also revealed that vitamin D supplementation did not change lipid or metabolic variables in CVD patients with metabolic syndrome [75].

The data on the effect of vitamin D supplementation on the incidence of cardiovascular events are also conflicting. In a recent randomized control trial, Manson *et al.* [23] reported that supplementation of vitamin D3 (2000 IU/day) in men and women older than 50 and 55 years, respectively, was not associated with the reduced incidence of cardiovascular events (myocardial infarction, stroke) nor decreased risk for death from cardiovascular causes. A recent umbrella review of meta-analyses by Khan *et al.*, [76] has also shown that vitamin D supplementation was not associated with the risk of cardiovascular mortality, myocardial infarction, coronary heart disease, or stroke. Similar conclusions have been drawn from a recent meta-analysis conducted on 83,000 patients, who were either treated with vitamin D or a placebo. Vitamin D supplementation did not lead to a significant decrease in the incidence of myocardial infarction, stroke, and cardiovascular mortality [77]. On the coregulates cardiovascular function, and there is an expression of VDR throughout the role significantly reduced the incidence of stroke in the Indian population [78]. A meta-analysis also concluded that reduced vitamin D levels are associated with an increased risk of ischemic stroke, but the effect of supplementation was not examined. Rodriguez *et al.* in their meta-analysis found that the vitamin D supplementation did not significantly improve arterial stiffness, assessed by pulse wave velocity and augmentation index [79]. Interestingly, patients with systemic lupus erythematosus supplemented with vitamin D and calcium have been shown to have increased arterial stiffness, compared with patients who were not supplemented [80].

Contradictory findings are reported in a recent clinical trial conducted on chronic kidney disease patients, indicating that vitamin D supplementation was associated with improved vascular compliance, assessed by endothelium-dependent brachial artery flow-mediated dilation, pulse wave velocity and circulating biomarkers (IL-6) [81].

## 8 VITAMIN D AND VASCULAR CALCIFICATION

Vascular calcification is characterized by calcium deposition in large and small blood vessels [19]. Calcium and phosphate depositions form hydroxyapatite crystals in blood vessels, similar to the ones in the bones, which can compromise the elasticity of the vessels and lead to stiffening. Vitamin D hypovitaminosis can also lead to abnormally increased vascular calcification, which is associated with an increased risk of atherosclerosis and stroke. Alterations in the calcium, phosphate, and vitamin D status in the body are associated with an increased incidence of vascular calcification. Findings have indicated that parathyroid hormone and fibroblast growth factor-23 (FGF23) play an important role in the etiology of vascular calcification. Vitamin D stimulates FGF23 activity in osteoblasts, whereas FGF23 inhibits the production of 1,25(OH)_2_D by down-regulating renal 1α-hydroxylase and up-regulating 24-hydroxylase. Therefore, FGF23 and klotho can modulate calcium and phosphate balance in the body, and suppression of FGF23 increases serum calcium and phosphate concentration [82].

### 8.1 Animal Studies

Animal studies have suggested that vitamin D hypervitaminosis, particularly elevated levels of 25(OH)D, can lead to calcium deposition in the aorta and coronary vessels [83]. Increased serum calcium and cholesterol levels were shown after a sublethal dose of vitamin D, 7.5 mg/kg. Moreover, a high dose of vitamin D was shown to induce calcification in animal models. Prolonged intake of vitamin D supplementation, even within the treatment of vitamin D deficiency, can lead to vitamin D intoxication, characterized by hypercalcemia, hyperphosphatemia, and organ damage [84, 85]. In a recent animal study, treatment of vitamin D was seen to be associated with increased vascular tone and thickness in cerebral arterioles, because of intense vasoconstriction and decreased endothelial nitric oxide production, leading to vascular dysfunction [86, 87].

### 8.2 Human Studies

Bover *et al.*, [88] raised a concern about the potential adverse effects of excessive calcium intake among old adults, particularly in those with chronic kidney disease, low bone mineral density, and vascular calcification, and advised that calcium-rich diets and high doses of vitamin D should be avoided. Indeed, the mentioned umbrella review of meta-analyses by Khan *et al.*, confirmed that combined calcium plus vitamin D supplementation increased the risk for stroke, but vitamin D supplementation alone was not associated. Additionally, during a 10-year follow-up in the Multi-Ethnic Study of Atherosclerosis (MESA), calcium supplementation use in older adults was associated with the increased risk for coronary artery calcification, but the dietary calcium related to the decreased risk [89].

## CONCLUSION

Cardiovascular diseases (CVD), particularly coronary heart disease (CHD), remain a leading cause of mortality worldwide, presenting complex interactions between genetic, lifestyle, and environmental factors. In an attempt to understand the intricate relationship between CHD death rates and potential contributors, researchers have turned their attention to geographical disparities and the role of factors such as vitamin D deficiency.

The intricate relationship between vitamin D deficiency and cardiovascular disease (CVD) mortality extends beyond biological factors and encompasses a broader spectrum of geographical disparities and health outcomes. The geographic distribution of CVD mortality rates as outlined in “The World Heart Report 2023” [90] underscores the multifaceted nature of this global health concern. The regions with the highest age-standardized CVD death rates, such as Central Europe, Eastern Europe, and Central Asia, and the North Africa and Middle East region, coincide with areas that have reported significant frequencies of vitamin D deficiency. This observation raises intriguing questions about the potential relationship between vitamin D deficiency and the higher prevalence of CVD mortality in these regions. Moreover, the observed disparities in progress across different regions in terms of CVD death rate reduction underscore the complex interplay between various factors, including healthcare accessibility, socio-economic conditions, and potentially vitamin D deficiency.

Our “Introduction” highlighted the lower prevalence of vitamin D deficiency among Europeans and Caucasians compared to regions like India, Pakistan, Afghanistan, and Tunisia. However, the high age-standardized CVD death rates in regions like Central Europe, Eastern Europe, and Central Asia, along with North Africa and the Middle East region, emphasize that vitamin D deficiency might not be the sole determinant of CVD mortality. The interplay of multiple factors, including genetic predisposition, lifestyle choices, dietary habits, healthcare infrastructure, and socio-economic conditions, can collectively contribute to the observed CVD mortality rates.

Vitamin D3 regulates cardiovascular function, and there is an expression of VDR throughout the cardiovascular system. The mechanisms of action can include regulation of innate and adaptive immune responses and inflammation; regulation of parathyroid hormone, calcium, and phosphate homeostasis; regulation of RAS, lipid levels, and insulin sensitivity. By all these mechanisms vitamin D can influence cardiac and arterial function, development of hypertrophy, fibrosis, atherosclerosis, endothelial dysfunction, hypertension, and vascular calcification. Numerous studies *in vitro*, in animals, and humans were performed on the effect of vitamin D on cardiovascular function. Studies based on animal models have revealed that vitamin D supplementations can have advantageous effects on some cardiovascular parameters, while on some others can have adverse effects. Studies in humans showed that the status of vitamin D can influence cardiovascular health and that vitamin D deficiency can be associated with some cardiovascular diseases. However, discrepancies in the results often exist. Moreover, discrepancies in the findings from the clinical studies have been reported on both the effects and the effectiveness of vitamin D supplementation. Hypervitaminosis D can also have adverse cardiovascular effects, which need clinical consideration as well. Seems that a clear link connecting adverse cardiovascular effects, vitamin D deficiency/surplus, and its therapeutic effects is still missing and requires more profound studies in the future.

## AUTHORS’ CONTRIBUTIONS

Dr. Abolfazl Zendehdel: conceptualized and designed the study, drafted the initial manuscript, reviewed, and revised the manuscript.

Dr. Ehsan Sekhavati Moghadam and Dr. Amir Shakarami: Designed the data collection instruments, collected data, carried out the initial analyses, reviewed, and revised the manuscript. Coordinated and supervised data collection, and critically reviewed the manuscript for important intellectual content.

## Figures and Tables

**Fig. (1):**
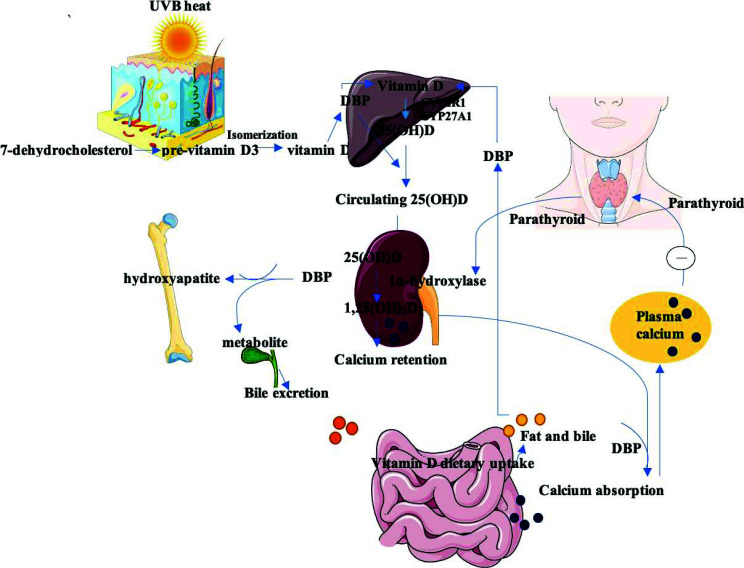
Vitamin D from the sun is absorbed in the skin and hydroxylated in the liver to 25(OH)D. Reduced calcium levels mediate the production of parathyroid hormone that leads to the conversion of 25(OH)D in the kidney to 1,25(OH)2D3. Expression of vitamin D receptors in several tissues, that mediates the absorption of calcium and phosphorous.

**Fig. (2):**
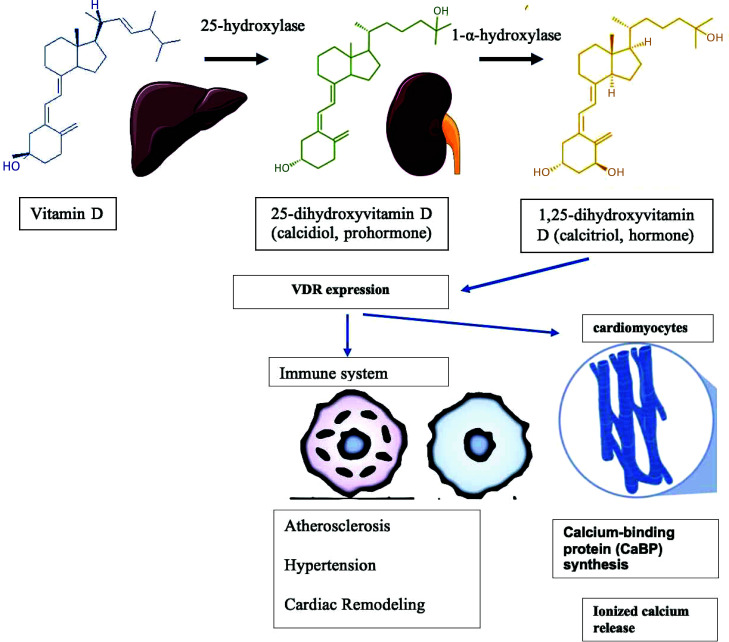
Structural modifications, following the metabolism of vitamin D into its active hormone form. This figure depicts the intricate relationship between vitamin D, immune system regulation, and cardiovascular diseases. The diagram illustrates how vitamin D, through its interaction with immune cells, can modulate inflammation, regulate immune responses, and impact cardiovascular health.

## LIST OF ABBREVIATIONS

CRP: C-reactive Protein
VDR: Vitamin D Receptor
RXR: Retinoid-X-receptor
MMP: Matrix Metalloproteinase
TGF-β: Tissue Growth Factor-beta
GADD45A: DNA-damage-inducible Protein 45 Alpha
NCOR1: Nuclear Receptor Co-repressor 1
PON2: Paraoxonases 2
HDL: High-density Lipoprotein
JNK2: c-Jun N-terminal Kinase 2
HSP-70: Heat Shock Protein-70
lnRNA: long Noncoding RNA
BNP: Brain Natriuretic Peptide
β-MHC: beta-myosin Heavy Chain
ANF: Atrial Natriuretic Factor
T reg: T regulatory
INF-γ: Interferon-gamma
Pin1: Prolyl Isomerase-1
SOD: Superoxide Dismutase
ROS: Reactive Oxygen Species
NO: Nitric Oxide
CFU-Fs: Colony-forming Units – Fibroblasts
RAS: Renin-angiotensin System
FMD: Flow-mediated Dilation
VC: Vascular Calcification
